# Expression of miR-21 and its targets (PTEN, PDCD4, TM1) in flat epithelial atypia of the breast in relation to ductal carcinoma in situ and invasive carcinoma

**DOI:** 10.1186/1471-2407-9-163

**Published:** 2009-05-28

**Authors:** Liqiang Qi, Joost Bart, Lu Ping Tan, Inge Platteel, Tineke van der Sluis, Sippie Huitema, Geert Harms, Li Fu, Harry Hollema, Anke van den Berg

**Affiliations:** 1 Department of Pathology & Medical Biology, Groningen University Medical Centre and University of Groningen, Groningen, The Netherlands; 2 Department of Breast Cancer Pathology and Research Laboratory, State Key Laboratory of Breast Cancer Research, Cancer Hospital of Tianjin Medical University, Tianjin, PR China

## Abstract

**Background:**

Flat epithelial atypia (FEA) of the breast is characterised by a few layers of mildly atypical luminal epithelial cells. Genetic changes found in ductal carcinoma in situ (DCIS) and invasive ductal breast cancer (IDC) are also found in FEA, albeit at a lower concentration. So far, miRNA expression changes associated with invasive breast cancer, like miR-21, have not been studied in FEA.

**Methods:**

We performed miRNA in-situ hybridization (ISH) on 15 cases with simultaneous presence of normal breast tissue, FEA and/or DCIS and 17 additional cases with IDC. Expression of the miR-21 targets PDCD4, TM1 and PTEN was investigated by immunohistochemistry.

**Results:**

Two out of fifteen cases showed positive staining for miR-21 in normal breast ductal epithelium, seven out of fifteen cases were positive in the FEA component and nine out of twelve cases were positive in the DCIS component. A positive staining of miR-21 was observed in 15 of 17 IDC cases. In 12 cases all three components were present in one tissue block and an increase of miR-21 from normal breast to FEA and to DCIS was observed in five cases. In three cases the FEA component was negative, whereas the DCIS component was positive for miR-21. In three other cases, normal, FEA and DCIS components were negative for miR-21 and in the last case all three components were positive. Overall we observed a gradual increase in percentage of miR-21 positive cases from normal, to FEA, DCIS and IDC. Immunohistochemical staining for PTEN revealed no obvious changes in staining intensities in normal, FEA, DCIS and IDC. Cytoplasmic staining of PDCD4 increased from normal to IDC, whereas, the nuclear staining decreased. TM1 staining decreased from positive in normal breast to negative in most DCIS and IDC cases. In FEA, the staining pattern for TM1 was similar to normal breast tissue.

**Conclusion:**

Upregulation of miR-21 from normal ductal epithelial cells of the breast to FEA, DCIS and IDC parallels morphologically defined carcinogenesis. No clear relation was observed between the staining pattern of miR-21 and its previously reported target genes.

## Background

Flat epithelial atypia (FEA) was first proposed as an entity in 2003 in the World Health Organization classification of tumours of the breast [1]. It is defined as an intraductal neoplasm, characterized by the replacement of original epithelial cells by a single or 3–5 thick layer of mildly atypical cells with loss of polarization. However recent insights show that FEA actually consists of dilated lobules, rather than dilated ducts [2]. With the wide application of mammography screening in women, FEA is more frequently detected since calcification is relatively common in FEA. Although FEA is intensively studied there is still disagreement on how to deal with it in clinical patient care, since only few patients in whom only FEA was diagnosed developed subsequent breast cancer [3,4]. Patients with ductal carcinoma in situ (DCIS), a known premalignant lesion that needs to be treated, show concurrent FEA in about 30% of patients. For that reason it is interesting to study whether FEA has to be regarded as a true premalignant lesion or only as an indolent precursor lesion, with or without relationship with DCIS.

Loss of heterozygosity on 3p, 9q, 10q, 11q, 17p, 17q, and recurrent gains on 15q, 16p and 19 have been observed in approximately 10 to 50% of FEA [5-7]. These genomic aberrations are also found in DCIS and invasive ductal carcinoma (IDC) and suggest that FEA might indeed be considered as a premalignant lesion, which can develop into DCIS or IDC. Recent insights in oncogenesis have revealed that micro-RNAs (miRNAs) can play crucial roles in malignant transformation acting as onco-miRNAs or tumour suppressor miRNAs [8].

MiRNAs are small noncoding single stranded RNAs of about 21–23 nucleotides, which negatively modulate protein expression by targeting mRNA transcripts and triggering either translation repression or RNA degradation [9-12]. MiR-21 (miR-21) is one of the most studied miRNAs in cancer and is highly upregulated in breast cancer as compared with normal tissue [13,14]. Mir-21 levels were higher in grade 2 and 3 as compared to grade 1 IDC [14].

So far, only a few miR-21 target genes have been identified in several cancer types including breast cancer. Zhu et al. showed that miR-21 targets the tumor suppressor gene, Tropomyosin 1 (TM1) [15]. Frankel et al[16] demonstrated that inhibition of Programmed Cell Death 4 (PDCD4) in MCF-7 cells significantly alleviated the anti-proliferative effect of miR-21 inhibition. Chan et al. showed that miR-21 acts as an anti-apoptotic factor in human glioblastoma cells [17]. In hepatocellular cancer miR-21 regulates the expression of the tumor suppressor gene phosphatase and tensin homolog (PTEN) [18].

Although several studies focus on the expression of miR-21 in breast cancer, there are no studies on the expression of miR-21 in FEA. We performed miRNA ISH, which allows accurate and direct detection of miR-21 in normal, FEA, DCIS and IDC breast tissue. The expression of the experimentally confirmed target genes, PTEN, PDCD4 and TM1 were studied in normal, FEA, DCIS and IDC.

## Methods

### Specimen collection and processing

Twenty-five patients with simultaneous FEA and DCIS were identified in a series of 270 cases of DCIS diagnosed from January, 2004 to December, 2006 in the Department of Breast Cancer Pathology and Research Laboratory, Cancer Hospital of Tianjin Medical University, Tianjin, China. Twenty-one IDC cases were selected from University Medical Center Groningen (UMCG), Groningen, The Netherlands. All of the specimens were fixed by 10% formalin and embedded in paraffin. Fifteen patients with simultaneously FEA and/or DCIS were selected for miR-21 RNA-ISH based on a strong and homogeneous positive staining for β-actin using RNA-ISH, indicating a good quality of RNA in the tissue blocks. Seventeen of the 21 IDC (including three stage 1, nine stage 2 and nine stage 3 cases) contained good quality RNA and also showed a homogenous staining pattern over the tissue sections as determined by performing β-actin RNA-ISH. This selection criterion is important for this study since we want to compare the staining intensities in different tissue components within the same tissue block. FEA was morphologically defined as described in the Background. Additionally its existence was immunohistochemically confirmed using a panel of cytokeratins (CK8/18, CK5/6 and CK14). Only a monomorphic and monotypic CK8/18 positive intraductal cell proliferation was regarded as FEA, whereas mixtures of CK8/18 positive cells together with CK5/6 or CK14 positive cells were regarded as (benign) ductal hyperplasia [1,2,19,19]. DCIS and invasive carcinoma were morphologically defined using standard criteria [1]. All study protocols were approved by the local medical ethical committee of the University Medical Centre Groningen. For the tissue specimens from China, protocols were approved by the Tianjin Medical University Administrative Panel on Human Subjects in Medical Research and the institutional Review Boards of Tjanjin Cancer Research institution.

### β-actin RNA-ISH

β-actin RNA-ISH was performed on routinely fixed paraffin-embedded tissue sections by use of standard laboratory protocols. In short, paraffin tissue sections were deparaffinized and air-dried for 10 min. Slides were treated with proteinase K (Roche, 15 μg/ml) at 37°C for 1 hr. After being washed with PBS, slides were incubated with 1 ng/μl anti-sense β-actin DIG-labeled probe (Roche, Mannheim, Germany) in a hybridization solution consisting of 5× Denhardt's solution, 2× SSC, 10% dextran sulphate, 30% formamide, 1 mg/ml t-RNA, and 2 mg/ml salmon sperm DNA, overnight at 55°C. After being washed, slides were treated with 10 mg/ml RNase (Sigma-Aldrich, Steinheim, Germany) at 37°C for 30 min, washed and incubated with anti-DIG-labeled alkaline phosphatase Fab fragments (Roche) for 1 hr in 0.1 M maleic acid buffer containing 0.15 M NaCl, 2% blocking buffer, and 1% Triton X-100. Staining reaction was performed overnight with 4-Nitro blue tetrazolium chloride (NBT) (Roche) and X-phosphate/5-Bromo-4-chloro-3indolyl-phosphate (BCIP) (Roche) in 50 mM Tris-HCl, 150 mM NaCl, 50 mM MgCl2 pH = 9.7 buffer. In all experiments a negative control, i.e. staining without β-actin probe, was included.

### miR-21 ISH

MicroRNA-ISH was performed on routinely fixed paraffin-embedded tissue sections. Paraffin tissue sections were deparaffinized with xylene, rehydrated with ethanol dilution series and treated with 15 μg/ml proteinase K (Roche) at 37°C for 15 min. After a washing step with 0.2% Glycine in PBS, slides were fixed with 4% formaldehyde and washed with phosphate buffered saline (PBS), and 2 × SSC. After drying, slides were incubated with hybridization buffer consisting of 50% formamide, 0.25 mg/ml salmon sperm DNA, 1 mg/ml t-RNA, 0.01 M Dithiothreitol (DTT) (Roche), 10× Denhardt's solution, 10% dextran sulphate, 4× SSC at 37°C for 2 hr. Then slides were hybridized with 20 nM DIG-labeled miR-21 probe (Exiqon, Copenhagen, Denmark) diluted in hybridization buffer at 51°C overnight. After washing, slides were treated with blocking buffer (2% sheep serum, 0.1% Tween in PBS) at 37°C for 30 min. Slides were incubated with anti-DIG-AP Fab fragments in blocking buffer at 37°C for 1 hr and washed with 0.1% Tween in PBS and AP buffer (100 mM Tris-HCl, 100 mM NaCl, 5 mM MgCl2, 0.05% Tween 20, pH = 9.5), miR-21 was visualized in a staining reaction with NBT/BCIP solution (4.5 μl NBT, 3.5 μl BCIP, 1 μl levamisol in 1 ml AP buffer). The last step was refreshed for 3 times over a period of 1 – 3 days until the staining was visible. In all experiments a negative control, i.e. staining without miR-21 probe, was included.

The slides were scored independently by two observers and positive cases were defined when more than 10% of the cells of interest showed a cytoplasmic staining. Discrimination of weak and strong positive staining was based on the intensity observed in different components within the same area of the tissue section to exclude variation in staining intensity caused by differences during the fixation procedure. In general, the signals for β-actin were more pronounced than the signals for miR-21, supporting our pre-selection criteria. However, in some cases miR-21 staining appeared to be more pronounced than the β-actin staining.

### IHC staining of PTEN, PDCD4 and TM1

Paraffin-embedded sections (3 μm) were deparaffinized, rehydrated and stained using routine laboratory protocols. Antigen retrieval and antibody dilutions are summarized in table 1. Endogenous peroxidase was blocked with 0.3% hydrogen peroxide in phosphate buffered saline (PBS) for 30 minutes and endogenous biotin was blocked using a Blocking Kit (Vector Laboratories, Burlingame, USA). The sections were incubated for 1 hour at room temperature with antibodies diluted in 1% BSA in PBS. The sections were subsequently incubated with secondary and tertiary antibodies (all from DAKO) for 30 minutes. The staining was visualized with 3,3'-diaminobenzidine as chromogen for 10 minutes and the slides were counterstained with hematoxylin. Normal breast tissue was used as a positive control for PTEN and TM1 and normal colon tissue for PDCD4 staining. The staining patterns for all three antibodies were compared to previously reported staining patterns in these tissues to assess the specificity of the staining [20-22]. For PTEN we used the same antibody as used previously for prostate cancer [23]. For PDCD4 and TM1 specificity of the antibodies was shown previously by Western blot [24,25]. For PDCD4 normal colonic epithelium served as positive and intestinal type colon carcinoma served as negative control tissue (additional file 1). For TM1 colon served also as control tissue, with the muscularis mucosa and the smooth muscle layer around capillaries as positive and the colonic epithelium as negative controls (additional file 1). Negative controls were obtained by omission of the primary antibodies from the staining procedure.

**Table 1:** Details of immunohistochemistry procedure

Antibody	clone (source)	dilution	Antigen retrieval
PTEN	6H2.1 (Cascade BioScience)	1:100	Microwave (10 mM sodium citrate pH = 6.0)

PDCD4	ab51495 (Abcam)	1:100	Microwave (10 mM sodium citrate buffer pH = 6.0)

TM1 (alpha)	ab55915 (Abcam)	1:200	Protease 0.1%, 30 min

The staining was scored independently by two observes and classified as: (-) negative staining; (+) weak positive staining of 10% or more of the cell type of interest; (++) moderate positive staining in 10% or more of the cell type of interest; (+++) strong positive staining of 10% or more of the cells of interest. PTEN demonstrated a nuclear staining, TM1 a cytoplasmic staining and PDCD4 showed both cytoplasmic and nuclear staining, which was scored independently.

### Statistical analysis

Statistical analysis for comparison between normal breast tissue, FEA, DCIS and IDC for positive rates of miR-21 was performed using 1-sided Fisher exact test or Chi-square test. P values less than 0.05 were considered significant.

## Results

### β-actin miRNA-ISH

Fifteen out of 25 tissue blocks showed a positive and homogeneous staining pattern for β-actin RNA-ISH which showed that the quality of the tissue specimens and the fixation procedure resulted in sufficiently high quality RNA for ISH throughout the whole tissue section. IDC showed a positive staining with β-actin in 17 out of 21 cases. Ten FEA and four IDC showed no or only a very weak and focal (restricted to the outer areas of the tissue and not in the central regions) signal for β-actin and were excluded due to bad quality RNA in the tissue block.

### miR-21 ISH

**Figure 1:**
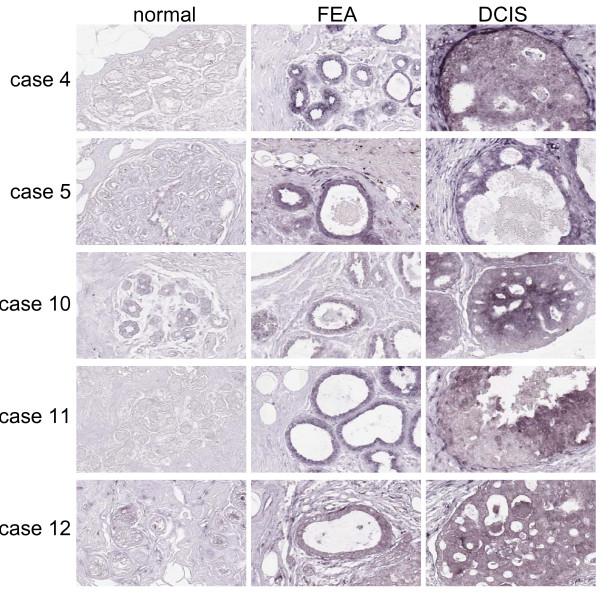
**Expression of miR-21 in 5 patients presenting simultaneously with normal breast epithelium, FEA, DCIS by RNA ISH**. The expression of miR-21 was detected predominantly in the cytoplasm in luminal cells. An increase in staining intensity for miR-21 was observed from normal, to FEA and DCIS in these 5 patients. Staining intensity in FEA and DCIS was similar in 4 out of 5 cases (200×).

**Figure 2:**
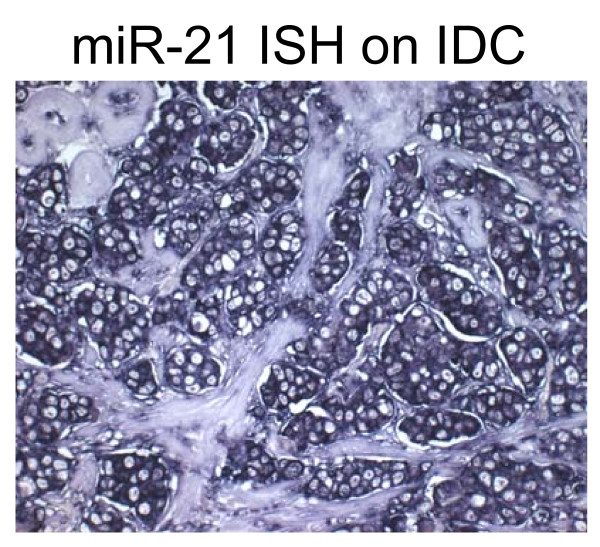
**Expression of miR-21 in IDC by RNA ISH**. A representative example of IDC showing strong cytoplasmic staining in the vast majority of the tumor cells.(200×).

We observed staining of miR-21 in the cytoplasm of normal breast epithelium in two out of 15 cases (13%). FEA was present in all cases and stained positive in seven cases (47%). For six of these seven cases, the FEA component stained positive for miR-21, whereas the normal component stained negative. DCIS was present in 12 cases and stained positive in nine cases (75%) (Table 2). In 12 of the samples, all three components were present in the tissue section. A marked increase in staining intensity was observed from normal breast to both FEA and DCIS in five cases (#4, 5, 10, 11, 15) (Fig. 1). In three cases (#1, 7 and 9) staining in FEA was negative, whereas DCIS stained positive, in three cases (#9, 12, 14) staining was negative in all three components and in one case staining was positive in all three components (#2). IDC showed a consistent strong positive signal in 15 out of 17 cases (88%) (Fig. 2). In general, the percentage of cases showing miR-21 expression was low in normal tissue (13%), and increased in FEA (47%), DCIS (75%), and IDC (88%). Using Fisher exact test, we found that the percentage of miR-21 positive cases was significantly higher in IDC and DCIS compared with normal breast tissue (P = 0.000 and P = 0.002, respectively). Moreover, an increase of the percentage of miR-21 positive cases was also observed in FEA (47%), although it did not reach significant levels (P = 0.054).

**Table 2:** Expression of miR-21 in normal, FEA and DCIS detected by RNA-ISH

Patient nr.	Normal breast	FEA	DCIS
1	-	-	±
2	+	+	+
3	-	+	na
4	-	+	+
5	-	+	+
6	-	-	na
7	-	-	+
8	+	-	+
9	-	-	-
10	-	±	+
11	-	+	+
12	-	-	-
13	-	-	na
14	-	-	-
15	-	+	+

Total	2/15 (13.33%)	7/15(46.67%)	9/12 (75%)

na, DCIS component not present in tissue block

### PTEN, TM1 and PDCD4 staining

**Figure 3:**
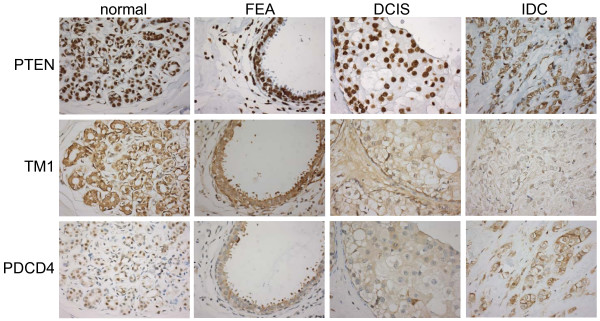
**Staining of three predicted miR-21 target genes in normal, FEA, DCIS and IDC tissue**. PTEN showed nuclear staining and was expressed strongly in normal, FEA, DCIS and IDC. TM1 expression in normal, FEA, DCIS and IDC showed cytoplasmic staining and there was a decrease from normal to FEA, DCIS and IDC. PDCD4 was expressed mainly in nuclei in normal breast while the staining pattern gradually shifted from nuclear to cytoplasmic in FEA, DCIS and IDC (400× magnification for all).

Normal breast tissue in all 15 cases showed a strong nuclear PTEN protein expression. The FEA, DCIS and IDC components also stained positive for PTEN albeit with varying intensities (Table 3, Fig. 3).

**Table 3:** Expression of miR-21, PTEN, PDCD4, TM1 immunohistochemistry staining in normal, FEA, DCIS and IDC

	miR-21			PTEN				TM1				PDCD4 (nuclear)				PDCD4 (cytoplasmic)			
					
Tissue type	-	±	+	-	+	++	+++	-	+	++	+++	-	+	++	+++	-	+	++	+++
Normal breast	13	0	2	0	0	0	15	0	4	10	0	4	2	4	5	13	1	1	0

FEA	8	1	6	0	3	5	7	0	2	10	3	1	0	6	8	13	0	2	0

DCIS	3	1	8	0	0	3	9	2	6	3	1	8	3	1	0	6	4	2	0

IDC	2	2	13	0	0	12	9	14	5	2	0	19	1	0	1	11	2	8	0

Cytoplasmic TM1 staining was seen in luminal cells in normal breast tissue in all cases. Similar to the normal breast tissue, FEA also showed positive staining in all cases. In DCIS staining was observed in ten out of 12 cases albeit at lower intensities as compared to normal and FEA. In IDC only seven out of 21 (33%) cases demonstrated a positive staining for TM1. In addition, TM1 protein expression was also seen in the cytoplasm of myoepithelial cells in some cases.

Staining of PDCD4 was seen in the nuclei and cytoplasm of luminal cells. The expression of PDCD4 was located predominantly in nuclei in normal breast tissue (73% positivity in nucleus versus 13% positivity in cytoplasm) and FEA (93% versus 13%), whereas in DCIS and IDC positive cytoplasmic staining was observed in approximately half of the cases and nuclear staining was decreased (Table 3, Fig 3).

### Direct comparison of miR-21 and target gene staining patterns

Based on the percentage of positive cases, an inversed trend can be observed for TM1 and miR-21, whereas, this is less evident for both PTEN and PDCD4 with miR-21. To further explore a possible inversed relation between the miR-21 staining pattern and the staining patterns, we also performed a case by case analysis. In IDC, TM1 staining is usually negative and miR-21 positive, whereas in normal tissue TM1 is usually positive and miR-21 negative. In FEA and DCIS there is a trend that the strongest TM1 signals were observed in the cases that lack miR-21 (additional file 2). For PTEN positive staining was observed in all four components (normal, FEA, DCIS and IDC), whereas a clear increase of miR-21 was observed for the percentage of positive cases from normal to FEA, DCIS and IDC (Additional file 3). For PDCD4 combination of the signal intensities for the nuclear and cytoplasmic staining with the miR-21 staining also showed no clear trend. However, the nuclear PDCD4 staining pattern does show an inverse relation to the miR-21 staining pattern for normal and IDC. For FEA and DCIS such a relation was not obvious (Additional file 4).

## Discussion

FEA is detected with increasing frequency, due to breast cancer screening programs. Although several studies indicate that FEA might be regarded as a premalignant lesion whether or not via progression to DCIS, clinical follow-up data did not unequivocally support this conclusion [3,4]. The present study confirms lack of miR-21 expression in the vast majority of the normal breast tissue samples and positivity in most IDC cases as previously reported [13,14]. FEA and DCIS express miR-21in 47 and 75% of the cases respectively.

Recent studies showed that at least some miRNAs act as oncogenes by playing a role in regulating proliferation and apoptosis. MiR-21 is consistently upregulated in invasive breast cancer, with a marked increase in grade 2/3 IDC as compared to grade 1 IDC cases [14]. In a single study, RNA-ISH using fluorescently labeled probes has been applied on breast cancer, demonstrating that miR-21 expression is frequently increased in IDC as compared with normal breast tissue [26]. In our study, a progressive increase of the percentage of positive cases of miR-21 was observed from normal (13%) to FEA (47%), DCIS (75%) and to IDC (88%). One exceptional case (#8) with positive staining in normal and DCIS, but not in the FEA component was observed. Based on the increase of number of positive cases from normal, to FEA, DCIS and IDC, it is tempting to speculate that increased miR-21 in FEA might be an indication of a pre-malignant phenotype of FEA. These findings are in line with previous studies that showed several genomic aberrations in a low percentage of FEA [5-7].

Thus far, two direct miR-21 target genes have been experimentally verified in breast cancer, i.e. TM1 [15] and PDCD4 [16,27,28]. A third potential miR-21 target gene, PTEN, was identified in hepatic cellular cancer and has not been tested in breast cancer, so far [18]. Direct effects of miR-21 function in breast cancer was obtained in two more recent studies by inhibiting miR-21, which resulted in reduced invasion and lung metastasis in MDA-MB-231 and suppressed cell growth of MCF-7 cells both in vitro and in a xenograft mouse model [27,28].

PTEN is a tumor suppressor gene, associated with negative regulation in the phosphoinositide-3-kinase (PI3 kinase) pathway [29]. Meng et al. showed that the expression of PTEN decreased in normal human hepatocytes after induction of miR-21. Moreover, using a luciferase reporter assay they demonstrated that PTEN is directly targeted by miR-21 [18]. In our study PTEN expression levels were +++ in all normal components, and ++ or +++ in the vast majority of FEA, DCIS and IDC compartments. The slight differences in staining intensities for PTEN in these components together with the strong increase observed for miR-21 from normal to IDC does not support a prominent role for miR-21 induced repression of PTEN in breast cancer. Moreover, matched analysis of miR-21 and PTEN per component and per case, also failed to show an inverse correlation. Previous studies have shown loss of PTEN protein expression in IDC [20,30] and in one study also in DCIS, albeit at a lower percentage of the cases as compared to IDC [31]. In our series no loss of PTEN was observed probably due to our relative small series.

PDCD4 was identified as a novel tumor suppressor gene and it was found to be downregulated in several types of human cancer. Frankel et al[16] demonstrated that miR-21 targeted PDCD4 in a luciferase based reporter assay. Moreover, they also demonstrated that downregulation of PDCD4 in MCF-7 cells significantly alleviated the anti-proliferative effect of miR-21 inhibition, which suggests an essential role for PDCD4 as a mediator of the biological effects of miR-21 in breast cancer cells. In breast cancer a decreased expression of PDCD4 was observed in comparison to normal breast tissue. Expression of PDCD4 was mainly localized in nuclei in DCIS while it was predominantly expressed in cytoplasm in normal breast tissue [32]. In contrast, we found that the PDCD4 expression shifted from nuclei to cytoplasm with the progression of breast tissue from normal to IDC, whereas nuclear expression of PDCD4 decreased from normal breast to FEA, DCIS and IDC. In colorectal cancer a shift from nuclear to cytoplasmic staining was observed from normal tissue, to colonic adenoma, and colorectal cancer [22] consistent with our findings. So far, the literature concerning the location of PDCD4 is still contradictory and there are no clear data demonstrating the specific function of either cytoplasmic or nuclear PDCD4. Since we only observed a shift from cytoplasm to nucleus or vice versa and not a marked change in the total amount of protein it is unlikely that PDCD4 is a direct target of miR-21. However, we can not exclude an indirect effect of miR-21 by regulating a protein that is involved in PDCD4 localization.

TM1 belongs to the family of tropomyosins (TMs) and acts as a suppressor of cellular transformation [33]. Targeting of TM1 by miR-21 was shown by the downregulation of TM1 expression upon induction of miR-21 and the upregulation of TM1 expression upon treatment with anti-miR-21 in a breast cancer cell line [15]. Our results showed that there was an obvious decrease in the percentage of positive cases for TM1 with progression, i.e. the majority of cases being positive in both normal and FEA and a minority of cases being positive in IDC. The staining pattern in normal and IDC shows an inversed relation to the miR-21 staining pattern consistent with targeting of TM1 by miR-21. For the FEA and DCIS components this inversed relation is less obvious.

In a recent paper, Talotta et al. showed that transcription of the primary miR-21 transcript is induced by AP-1 in response to RAS in the RAS inducible cell line FRTL-5/ER-RAS [34]. In this cell line model induction of miR-21 was responsible for the downregulation of PDCD4 and PTEN. No decrease was observed for TM1 protein levels. These data are not consistent with our staining results showing a consistently high expression level for PDCD4 and PTEN in normal, FEA, DCIS and IDC. In contrast, the staining pattern for TM1 shows an inversed staining pattern as compared to the miR-21 pattern. Whether this model is relevant for the situation in breast cancer development is questionable.

## Conclusion

The percentage of miR-21 positive cases gradually increased from normal to FEA, DCIS and IDC. Our results demonstrate that in five out of 12 cases with simultaneous presentation of normal, FEA and DCIS, the FEA and DCIS components share a similar miR-21 profile. This is consistent with previous publications showing similar genetic aberrations in FEA and DCIS. An inversed staining pattern was observed for miR-21 and TM1 in the normal and IDC components, but not in FEA and DCIS. These findings might support targeting of TM1 by miR-21 in breast cancer.

## Competing interests

The authors declare that they have no competing interests.

## Authors' contributions

GH, LPT and SH carried out the RNA-ISH. TS and IP performed immunohistochemistry stainings. AB supervised the miRNA ISH experiments. HH and JB were responsible for scoring of the stainings. LF participated in collection of material and LQ was in charge of data collection, statistical analysis and manuscript preparation. HH, JB and AB were responsible for the study design and for the preparation of the manuscript. All authors read and approved the final manuscript.

## Pre-publication history

The pre-publication history for this paper can be accessed here:

http://www.biomedcentral.com/1471-2407/9/163/prepub

## Supplementary Material

Additional file 1 **Postive and negative control of TM1 and PDCD4 in colon tissue**. PDCD4 staining in normal colonic epithelium (positive control) and in intestinal type colon carcinoma (negative control). Tropomyosin 1 staining in colon, smooth muscle of the muscularis mucosae and of the capillary wall as positive control, whereas the epithelium and lymphoid cells as negative control. Click here for file

Additional file 2 **Comparison of miR-21 and TM1**. Direct comparison of miR-21 and TM1 expression in different tissue components for each case separately. Click here for file

Additional file 3 **Comparison of miR-21 and PTEN**. Direct comparison of miR-21 and PTEN expression in different tissue components for each case separately. Click here for file

Additional file 4 **Comparison of miR-21 and PDCD4**. Direct comparison of miR-21 and PDCD4 expression in different tissue components for each case separately. Click here for file
