# Supplementary material for: Expression of miR-21 and its targets (PTEN, PDCD4, TM1) in flat epithelial atypia of the breast in relation to ductal carcinoma in situ and invasive carcinoma
Source: BMC Cancer. 2009 May 28;9:163. doi: 10.1186/1471-2407-9-163 (PMC2695476; doi:10.1186/1471-2407-9-163)
Supplement: Additional file 1 — Postive and negative control of TM1 and PDCD4 in colon tissue. PDCD4 staining in normal colonic epithelium (positive control) and in intestinal type colon carcinoma (negative control). Tropomyosin 1 staining in colon, smooth muscle of the muscularis mucosae and of the capillary wall as positive control, whereas the epithelium and lymphoid cells as negative control. [file 1471-2407-9-163-S1.ppt]

## Slide 1
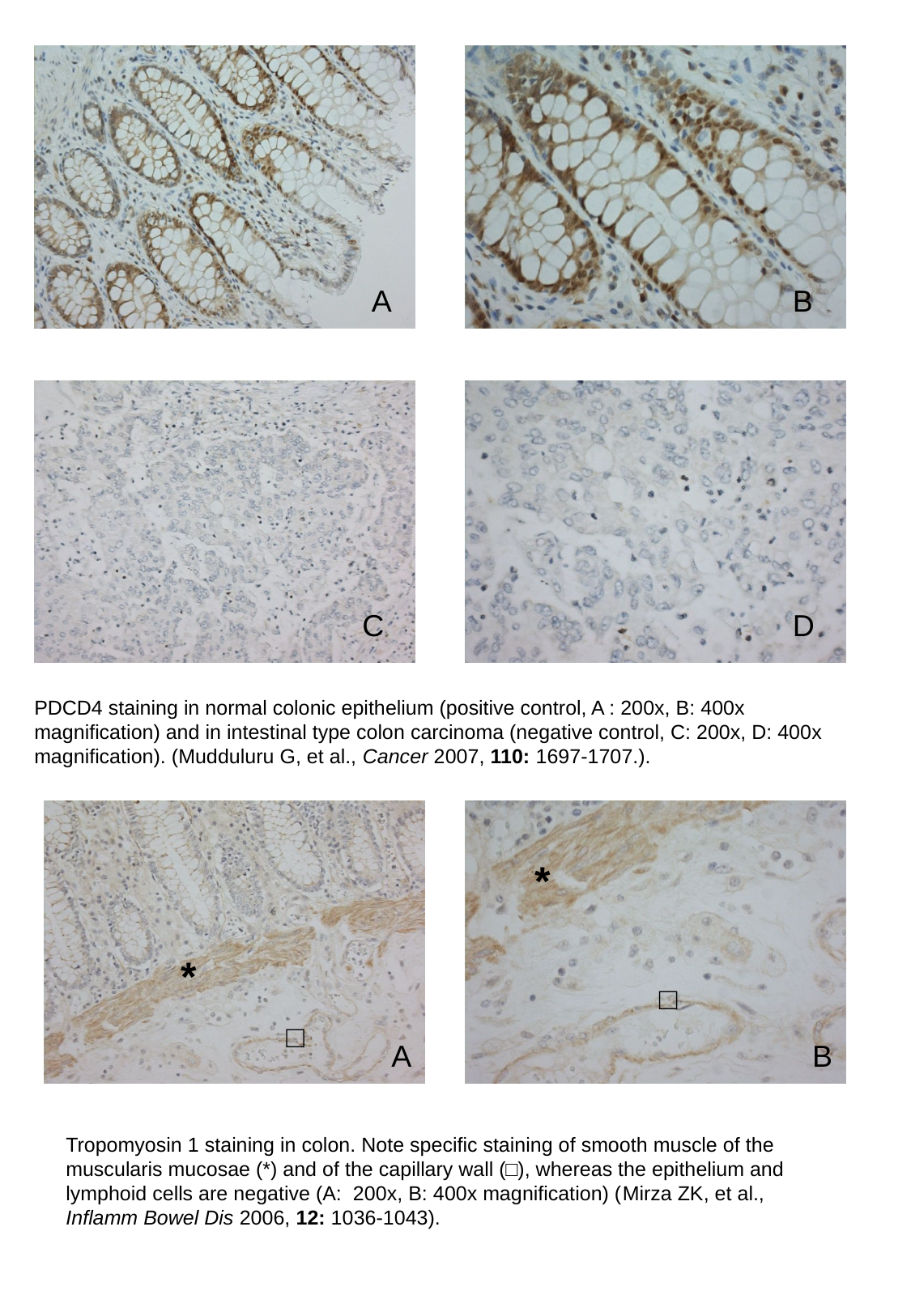

A
B
C
D
PDCD4 staining in normal colonic epithelium (positive control, A : 200x, B: 400x magnification) and in intestinal type colon carcinoma (negative control, C: 200x, D: 400x magnification). (Mudduluru G, et al., Cancer 2007, 110: 1697-1707.).
*
*
□
□
A
B
Tropomyosin 1 staining in colon. Note specific staining of smooth muscle of the muscularis mucosae (*) and of the capillary wall (□), whereas the epithelium and lymphoid cells are negative (A: 200x, B: 400x magnification) (Mirza ZK, et al., Inflamm Bowel Dis 2006, 12: 1036-1043).
